# A Computational and Spectroscopic Analysis of Solvate Ionic Liquids Containing Anions with Long and Short Perfluorinated Alkyl Chains

**DOI:** 10.3390/molecules29092071

**Published:** 2024-04-30

**Authors:** Karina Shimizu, Adilson Alves de Freitas, Jacob T. Allred, Christopher M. Burba

**Affiliations:** 1 Centro de Química Estrutural, Institute of Molecular Sciences, Instituto Superior Técnico, Universidade de Lisboa, Av. Rovisco Pais, 1049-001 Lisboa, Portugal; karina.shimizu@tecnico.ulisboa.pt; 2 Department of Natural Sciences, Northeastern State University, 611 N Grand Ave., Tahlequah, OK 74464, USA; allredj@nsuok.edu

**Keywords:** solvate ionic liquid, molecular dynamics simulations, vibrational spectroscopy, apolar–polar structural heterogenity, charge organization, vibrational mode assignments

## Abstract

Anion-driven, nanoscale polar–apolar structural organization is investigated in a solvate ionic liquid (SIL) setting by comparing sulfonate-based anions with long and short perfluorinated alkyl chains. Representative SILs are created from 1,2-bis(2-methoxyethoxy)ethane (“triglyme” or “G3”), lithium nonafluoro-1-butanesulfonate, and lithium trifluoromethanesulfonate. Molecular dynamics simulations, density functional theory computations, and vibrational spectroscopy provide insight into the overall liquid structure, cation–solvent interactions, and cation–anion association. Significant competition between G3 and anions for cation-binding sites characterizes the G3–LiC_4_F_9_SO_3_ mixtures. Only 50% of coordinating G3 molecules form tetradentate complexes with Li^+^ in [(G3)_1_Li][C_4_F_9_SO_3_]. Moreover, the SIL is characterized by extensive amounts of ion pairing. Based on these observations, [(G3)_1_Li][C_4_F_9_SO_3_] is classified as a “poor” SIL, similar to the analogous [(G3)_1_Li][CF_3_SO_3_] system. Even though the comparable basicity of the CF_3_SO_3_^−^ and C_4_F_9_SO_3_^−^ anions leads to similar SIL classifications, the hydrophobic fluorobutyl groups support extensive apolar domain formation. These apolar moieties permeate throughout [(G3)_1_Li][C_4_F_9_SO_3_] and persist even at relatively low dilution ratios of [(G3)_10_Li][C_4_F_9_SO_3_]. By way of comparison, the CF_3_ group is far too short to sustain polar–apolar segregation. This demonstrates how chemically modifying the anions to include hydrophobic groups can impart unique nanoscale organization to a SIL. Moreover, tuning these nano-segregated fluorinated domains could, in principle, control the presence of dimensionally ordered states in these mixtures without changing the coordination of the lithium ions.

## 1. Introduction

Ionic liquids (ILs) are a class of salts that exist in the liquid phase at or near room temperature. Interest in ILs is motivated, in part, by the unique property combinations available from a completely ionic system. For example, it is relatively easy to make an IL that has high ionic conductivity, a wide electrochemical window, and negligible vapor pressure. However, replicating this set of properties with “traditional” molecular compounds is quite challenging. Tailoring ILs towards specific property combinations requires an understanding of how their liquid-phase structure emerges from long-range Coulombic and short-range repulsive forces [1]. These interactions produce highly interconnected, charge-organized structures with distinct alterations in cation–cation and cation–anion radial distribution functions [2,3,4,5,6,7]. An additional source of the liquid-phase structure comes from polarity differences within the ions themselves. For example, increasing the length of the aliphatic side chain on 1-alkyl-3-methylimidazolium promotes the self-assembly of the hydrophobic regions [8,9,10,11,12,13,14,15,16]. Aggregation of these moieties causes further structural organization of the IL into polar and apolar domains. Similar nanoscale organization may also be driven by the anion when sufficiently long fluorinated alkyl chains are employed [17,18,19,20].

ILs are typically classified according to the nature of the constituent ions comprising the material. One early classification scheme divides ILs into protic, aprotic, and zwitterionic groups [21]. However, additional classes have emerged with the advent of new chemical varieties [22]. Solvate ionic liquids (SILs) are an intriguing IL subclass [23,24,25,26,27,28,29,30]. These materials are the product of an inorganic salt and a molecular solvent capable of coordinating the salt’s cation. The result is a completely ionic solution that consists solely of complexed cations and dissociated anions. Methyl-capped ethylene glycols, such as triethylene glycol dimethyl ether (triglyme, G3) or tetraethylene glycol dimethyl ether (tetraglyme, G4), are natural choices for the chelating agents due to their high affinity for Li^+^ ions. In those cases, polydentate coordination by linear glyme molecules causes the solvent molecule to wrap the cation in a manner that resembles cation coordination by crown ethers [26,29,31,32,33].

Creating an SIL requires a delicate balance of cation–solvent and cation–anion interactions [24,25]. Strong cation–solvent interactions are needed to eliminate uncoordinated and partially coordinated solvent molecules, while weak ionic interactions are required to minimize cation and anion associations. The relative basicity of the anion compared to the solvent molecule is a key factor in determining the overall SIL liquid structure [26]. For example, the prototypical “good” SIL [Li(G4)_1_][TFSI], where LiTFSI is lithium bis(trifluoromethanesulfonyl)imide, has few uncoordinated solvent molecules. MD simulations show that approximately 60% of Li–G4 complexes exist in a 1:1 ratio. An additional ~20% of the G4 participates in [Li(G4)_2_]^+^ or [Li_2_(G4)]^2+^ complexes, with ~15% more G4 forming poly-nuclear [Li*_x_*(G4)*_y_*]*^x^*^+^ complexes with *x* + *y* = 4. Replacing the weakly coordinating TFSI^−^ anion with NO_3_^−^ dramatically alters the Li–G4 interactions, increasing the fraction of G4 molecules that lack Li···O contacts.

**Scheme 1:**
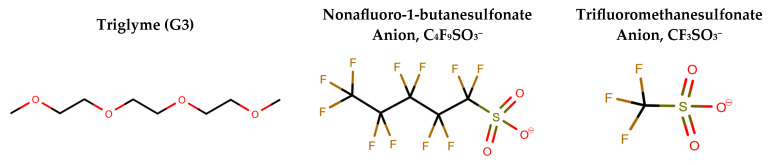
Structures of the G3 solvent molecule and the two anions that are evaluated. Atom coloring is as follows: fluorine (brown), sulfur (green), and oxygen (red). Carbon and hydrogen atoms are not explicitly shown.

While the influence of anion basicity on the overall liquid structure is well established in SILs, much less is known about how the size of fluorinated alkyl groups attached to the anion drives structural organization in an SIL context. Specifically, how are overall liquid structure, cation–solvent, and cation–anion interactions affected by asymmetrically lengthening a fluorinated alkyl side chain on the anion? We address this question by comparing two SIL systems created from triglyme, lithium trifluoromethanesulfonate (LiCF_3_SO_3_, anions with short fluorinated tails), and lithium nonafluoro-1-butanesulfonate (LiC_4_F_9_SO_3_, long fluorinated tail). Chemical structures for these species are given in Scheme 1. A combination of MD simulations, density functional theory calculations, and vibrational spectroscopy is employed to understand how the fluorinated side chain length drives SIL structure.

## 2. Results and Discussion

### 2.1. Structure of G3–LiC_4_F_9_SO_3_ and G3–LiCF_3_SO_3_ Mixtures

**Figure 1:**
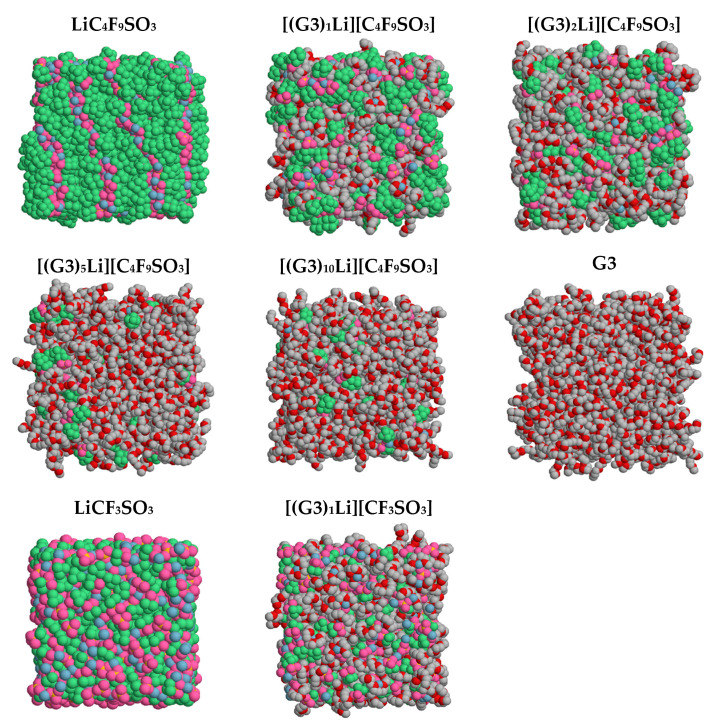
Simulation snapshots of the [(G3)*_n_*Li][C_4_F_9_SO_3_] mixtures with *n* = 1, 2, 5, and 10, [(G3)_1_Li][CF_3_SO_3_], LiCF_3_SO_3_, LiC_4_F_9_SO_3_, and pure G3. The blue color represents Li^+^ ions; the colors pink, yellow, green, and dark grey denote the O, S, F, and C atoms of the anions, respectively; and the colors red and light grey represent the O and C atoms of the triglyme molecule, accordingly.

Figure 1 presents simulation snapshots for different compositions of the [(G3)*_n_*Li][C_4_F_9_SO_3_] mixtures. Hydrophobic interaction among the C_4_F_9_ groups causes pure LiC_4_F_9_SO_3_ to form distinct polar and nonpolar regions that percolate throughout the simulation box. Strands of Li^+^ ions and SO_3_ end groups of the anion generate polar domains with a lamellar structure (Figure S1). The nonpolar subphase is composed of fluoroalkyl moieties. Mixing LiC_4_F_9_SO_3_ with G3 disrupts the mesostructural organization inherent to LiC_4_F_9_SO_3_, leading to a complex liquid phase mediated by cation–solvent, cation–anion, and polar–apolar interactions. The smaller CF_3_SO_3_^−^ anion lacks a large enough fluorinated group to support polar–apolar domain segregation in both high-temperature LiCF_3_SO_3_ and [(G3)_1_Li][CF_3_SO_3_].

**Figure 2:**
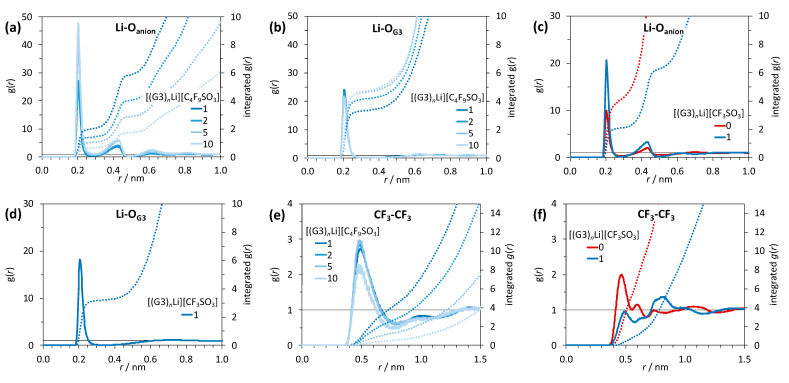
Radial distribution functions, *g*(*r*)s, between Li^+^ ions and O atoms in [(G3)*_n_*Li][C_4_F_9_SO_3_] and [(G3)*_n_*Li][CF_3_SO_3_] mixtures, with different compositions. (**a**,**c**) O atoms of the sulfonate group, (**b**,**d**) O atoms of the ether group, (**e**) terminal C atoms of the C_4_F_9_SO_3_^−^ anion, and (**f**) terminal C atoms of the CF_3_SO_3_^−^ anion. The dotted lines represent the integrated *g*(*r*).

Despite their simplicity, radial distribution functions, *g*(*r*)s, are a fundamental tool for understanding the local structure of liquids. The *g*(*r*)s extracted from the MD trajectories provide the probability of finding a pair of selected sites at a given distance. From their maxima and minima, one can infer preferential atomic positions and define the microstructural pattern of a condensed phase. Furthermore, the Fourier transform of *g*(*r*) yields the total scattering functions, which give the X-ray or neutron diffraction profiles when weighted by the appropriate factor. The *g*(*r*)s between the Li^+^ ions and O atoms of C_4_F_9_SO_3_^−^ and G3 are provided in Figure 2. Both cases show a sharp first peak located at 0.20 nm. Two less intense peaks appear in the C_4_F_9_SO_3_–Li *g*(*r*) functions at 0.43 and 0.62 nm. These additional features correspond to the second and third solvation shells centered on the Li^+^ ion, respectively. In contrast, the Li–G3 *g*(*r*) functions show a featureless second band near 0.65 nm. Integration of *g*(*r*) gives the number of neighboring atoms surrounding the central Li^+^ ion as a function of distance. These results are presented as dotted lines in Figure 2. Lithium ions are tetrahedrally coordinated by O atoms from the C_4_F_9_SO_3_^−^ anion in LiC_4_F_9_SO_3_ when the coordination shell boundary is placed at ca. 0.30 nm. As the G3 content increases, the integrated *g*(*r*) functions reveal a gradual replacement of anions by G3 molecules in the vicinity of the cation. With respect to the fluorobutyl part of the anion, the *g*(*r*) functions between the terminal C atoms of the anion produce the peak at 0.49 nm. If the end of the first solvation shell is placed at ca. 0.8 nm, the integrated *g*(*r*) function shows a reduction in the number of neighboring anion tails from ~10 in the pure salt to ~0.50 in [(G3)_10_Li][C_4_F_9_SO_3_]. This emphasizes the overall separation of the anion species as G3 is introduced into the system.

Radial distribution functions for [(G3)_1_Li][CF_3_SO_3_] are also presented in Figure 2 to explore the role of anion tail size on the liquid structure of these systems. The *g*(*r*) profiles for Li-O_anion_ and Li-O_G3_ are quite similar in [(G3)_1_Li][CF_3_SO_3_] and [(G3)_1_Li][C_4_F_9_SO_3_]. For example, the first sharp peak in the Li–O_anion_ *g*(*r*) function is 0.20 nm, and the average coordination number around Li^+^ is approximately four. The *g*(*r*) data relating distances between the C atoms of the CF_3_SO_3_^−^ ion is somewhat different from the C_4_F_9_SO_3_^−^ anion. The smaller trifluoromethyl group in CF_3_SO_3_^−^ causes a displacement in the *g*(*r*) maxima from 0.47 to 0.81 nm for LiCF_3_SO_3_ and [(G3)_1_Li][CF_3_SO_3_], respectively. A similar shift is not observed in the corresponding [(G3)*_n_*Li][C_4_F_9_SO_3_] series.

**Figure 3:**
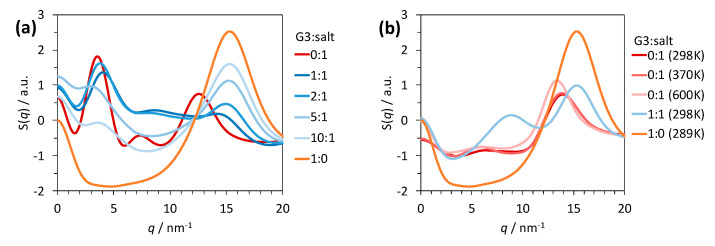
X-ray total structure factors, *S*(*q*). (**a**) LiC_4_F_9_SO_3_ at 370 K and [(G3)*_n_*Li][C_4_F_9_SO_3_] mixtures with different compositions at 298 K, and (**b**) LiCF_3_SO_3_ at different temperatures and the [(G3)_1_Li][CF_3_SO_3_] mixture at 298 K.

X-ray total structure factors are displayed in Figure 3 for scattering vectors *q* up to 20 nm^−1^. Pure LiC_4_F_9_SO_3_ exhibits a pre-peak in the low-*q* region (2 ≤ *q*/nm^−1^ ≤ 6), which is a characteristic of segregated polar and apolar domains. This so-called first sharp diffraction peak indicates breaking of the overall charge-ordering homogeneity due to intramolecular polarity differences within the ions. The result is the self-assembly of ions into mesoscopic structural motifs characterized by complex ionic disorder and charge confinement. By way of comparison, nanoscale structural organization is present for intermediate chain lengths of the 1-alkyl-3-methylimidazolium cation, and cations containing butyl side chains are on the cusp of polar–apolar structural organization [9,11]. The intermediate peak located between 6 and 10 nm^−1^ arises from cation–cation and anion–anion distances within the polar network, while the peak at a larger *q* value (10 ≤ *q*/nm^−1^ ≤ 20) accounts for a multitude of correlations between adjacent atoms (direct contact or adjacency peak). Total structure factor data, *S*(*q*), indicate the restoration of the global charge-ordering homogeneity and dilution of the same-charge correlations as the G3 content in the mixture increases. Also, the displacement of the direct contact peak to higher reciprocal distances (i.e., to shorter distances in direct space) is the outcome of the decrease in concentration of the bulky F atoms.

**Figure 4:**
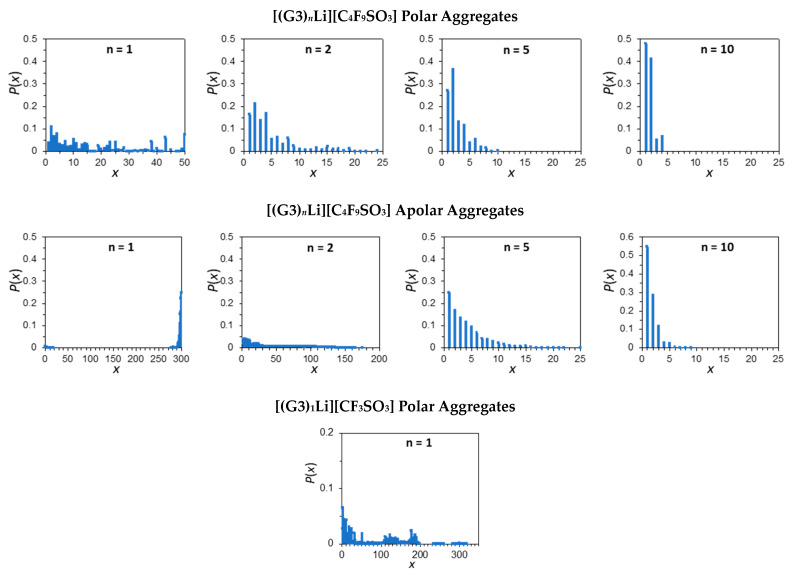
Probability distribution functions *P*(*x*) of polar and apolar aggregates involving Li^+^ ions and the SO_3_^−^ portion of the anion in [(G3)*_n_*Li][C_4_F_9_SO_3_] and [(G3)_1_Li][CF_3_SO_3_] mixtures (*n* = 10, 5, 2, and 1). The LiCF_3_SO_3_ system does not form apolar domains.

The size of the polar and apolar domains may be determined from probability distribution functions that measure different aggregate sizes; these results are visualized through histograms in Figure 4. Polar domains of the type Li^+^—SO_3_^−^—Li^+^—SO_3_^−^ are found in all of the systems; however, the size of these domains depends on the relative amount of G3 and the length of the anion’s fluorinated tail. For instance, the polar domains contain up to 50 units in [(G3)_1_Li][C_4_F_9_SO_3_], but these domains are broken into smaller-sized aggregates when the G3 content increases to [(G3)_10_Li][C_4_F_9_SO_3_] where the maximum cluster sizes equal four units. Nonpolar domains composed of the fluoroalkyl groups of the anion are also analyzed. The aggregate distributions indicate the presence of small clusters of anions (up to nine units) even in [(G3)_10_Li][C_4_F_9_SO_3_]. This underscores the affinity of the fluorobutyl groups to form supramolecular networks through their hydrophobic interactions.

The CF_3_ portion of the CF_3_SO_3_^−^ anion is too short to support polar–apolar structural organization, and the [(G3)_1_Li][CF_3_SO_3_] compound is characterized by a global charge ordering that permeates throughout the bulk phase. This is evident from the absence of a first sharp diffraction peak in the low-*q* region of the *S*(*q*) plots and probability distributions that have nearly all of the LiCF_3_SO_3_ in the MD simulation box participating in polar domain clusters (up to 300 units). Charge alternation is a feature of room-temperature molten salts. The lack of the charge-ordering peak is most likely a consequence of complete interference cancelation of peaks (same-charge correlations) and anti-peaks (different-charge correlations) [34,35,36]. Interestingly, the G3 molecule plays a different role in [(G3)_1_Li][CF_3_SO_3_] compared to [(G3)_1_Li][C_4_F_9_SO_3_]. Adding G3 increases the polar part, which introduces some overall charge ordering and attenuates the charge-ordering peak in *S*(*q*) for the [(G3)*_n_*Li][C_4_F_9_SO_3_] mixtures. The increase in polar parts of the [(G3)_1_Li][CF_3_SO_3_] system breaks this global charge ordering, resulting in a more prominent intermediate peak in the 1:1 mixture.

### 2.2. Coordinative Interactions between G3 and Li^+^ Ions

A connectivity analysis for pure LiC_4_F_9_SO_3_ and its mixtures with G3 was carried out to clarify the environment around the Li^+^ ions. These results are presented in Table 1. The Li–O_i_ parameter indicates the number of O atoms of species *i* coordinated with the cation, while Li-*i* represents the number of those species coordinated to the same Li^+^ cation. In the pure salt, the Li-O_anion_ and Li-anion data place the average number of anions coordinating a central Li^+^ ion at slightly fewer than four, and the anions predominantly interact with Li^+^ via a single O atom from each sulfonate group. The slightly larger Li-O_anion_ values in comparison to Li-anion point to a small contribution of bidentate binding of the anions to the cation. Monodentate anion coordination prevails in all studied compositions.

There is one G3 molecule in the first solvation shell of Li^+^ for mixtures up to [(G3)_5_Li][C_4_F_9_SO_3_]. G3 molecules are relatively bulky, and it is difficult to accommodate a second G3 molecule around the small cation. Moreover, G3 coordination must overcome the Coulombic attraction between ion pairs to break the cation free from the anion. Solvation shells with two G3 molecules are more prevalent in [(G3)_10_Li][C_4_F_9_SO_3_], where the fraction of free anions (and thus solvent-separated ion pairs) reaches 50%. Overall, the same observations for [(G3)*_n_*Li][C_4_F_9_SO_3_] mixtures are valid for [(G3)_1_Li][CF_3_SO_3_]. In pure LiCF_3_SO_3_, there are four anions coordinated by one O atom to the central cation, with a small contribution of bidentate binding. In the 1:1 mixture, the MD simulations reveal one G3 molecule and two CF_3_SO_3_^−^ ions in the first solvation shell of the cations.

**Figure 5:**
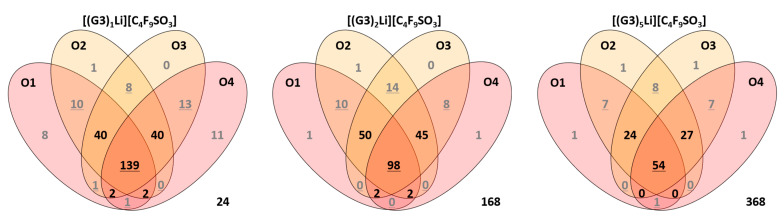

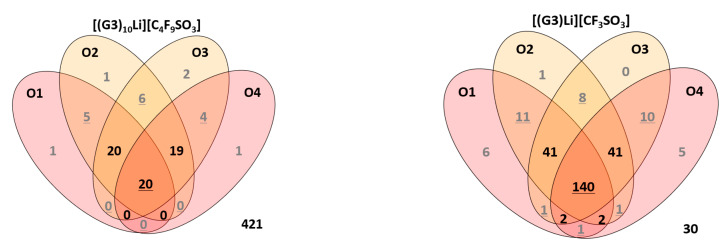
Venn diagrams depicting the connectivity between lithium ions and oxygen atoms of G3 molecules for [(G3)*_n_*Li][C_4_F_9_SO_3_] and [(G3)_1_Li][CF_3_SO_3_] at 298 K. The pink and yellow ellipses correspond to the outermost and inner oxygen atoms of the G3 molecules, respectively. The number of free, noninteracting G3 molecules in each mixture is shown in the bottom right (underlined black numbers = 4 ligating atoms; black numbers = 3 ligating atoms; underlined grey numbers = 2 ligating atoms; grey numbers = 1 ligating atom).

Venn diagrams depicting the connectivity between Li^+^ ions and O atoms of G3 molecules at 298 K are shown in Figure 5. The threshold used to account for the connectivity was the end of the first peak in *g*(*r*) Li-O_G3_ (ca. 0.275 nm) [26]. As expected, the total number of G3 molecules that interact with Li^+^ ions and the number of O atoms per G3 molecule bound to those ions are sensitive to sample composition. For example, 15.8% of G3 molecules coordinate Li^+^ in the [(G3)_10_Li][C_4_F_9_SO_3_] sample, and only 25.3% of those molecules experience tetradentate coordination. The presence of a second G3 molecule around the cation results in a larger tridentate population size. Changing the composition to [(G3)_5_Li][C_4_F_9_SO_3_] raises the overall fraction of “interacting” G3 molecules to 26.4% as well as the number of tetradentate interaction motifs to 40.9%. Further increases in LiC_4_F_9_SO_3_:G3 ratios to 1:2 and 1:1 increase the percentage of tetradentate G3 among coordinating G3 molecules to 42.4% and 50.3%, respectively. Shortening the fluorinated tail has a marginal impact on tetradentate G3 population sizes. For instance, tetradentate G3 comprises 51.9% of the coordinating G3 molecules in the [(G3)_1_Li][CF_3_SO_3_] mixture.

**Figure 6:**
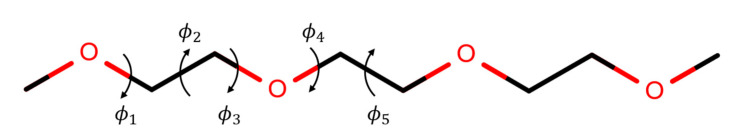
Numbering sequence used in distribution analyses of the G3 molecule’s torsion angles (curved arrows).

MD simulations also reveal an alteration in the G3 conformation upon cation coordination. Figure 6 portrays the numbering sequence adopted for the G3 molecule to aid in the discussion of the molecular conformation. Given the symmetry of the G3 moiety, the equivalent dihedral angles of both halves of the molecule were analyzed together. The averaged distribution analyses of torsion angles are provided in Figures S2 and S3. When referring to dihedral angles, we use the following notation: g^±^ for gauche (±30° to ±90°) and t for trans (150° to 210°).

For the dihedrals of the type COCC, Li–O binding increases the trans population size when moving from pure G3 to [(G3)_1_Li][C_4_F_9_SO_3_] (ca. 14% for $\phi_{1}$ and 8% for $\phi_{2}$ and $\phi_{3}$). The OCCO portion of the molecule is predominantly in a gauche conformation for pure G3, and binding with Li^+^ further increases the population size of gauche conformers at the expense of the trans conformers. For example, there are reductions of ca. 72% and 87% in trans conformers for $\phi_{4}$ and $\phi_{5}$, respectively, in [(G3)_1_Li][C_4_F_9_SO_3_]. Additionally, these dihedrals are displaced to smaller angles.

Complexes between lithium salts and oligomeric ethylene oxides have been extensively studied due to their relevance in lithium battery applications, and it is helpful to compare our MD simulation results with several noteworthy cases. Poly(ethylene oxide) and short-chain ethylene oxide oligomers frequently adopt helical conformations of the (tgt)*_n_* type [37,38,39,40]. Lithium ions are six-fold coordinated in the (G3)_1_LiN(SO_2_CF_3_)_2_ crystal. Each Li^+^ is coordinated by the four O atoms of a G3 molecule as well as two O atoms attached to separate S atoms of a single N(SO_2_CF_3_)_2_^−^ anion [41]. Thus, cations and anions form bidentate contact ion pairs in this solvate structure with the anion arranged in a cisoid conformation. The G3 molecule adopts a tg^−^t.tg^+^g^+^.tg^+^t sequence of torsion angles. The (G3)_1_(LiCF_3_SO_3_)_2_ and (G3)_1_LiN(SO_2_C_2_F_5_)_2_ compounds both exhibit five-fold Li^+^ interaction motifs. In (G3)_1_LiN(SO_2_C_2_F_5_)_2_, Li^+^ ions interact with all four O atoms of a single G3 molecule plus a single O atom from the anion. This coordination environment produces a g^−^g^−^t.tg^+^g^+^.tg^+^t torsion angle sequence for G3 [42]. This is contrasted with (G3)_1_(LiCF_3_SO_3_)_2_, where Li^+^ interacts with three O atoms from G3 and two O atoms from separate CF_3_SO_3_^−^ anions. The G3 molecule adopts the familiar tg^+^t.tg^−^t.tg^+^t conformation [43]. Similar interaction motifs are found in liquid and polymeric systems [44,45,46], including [(G3)_1_Li][N(SO_2_CF_3_)_2_] and [(G4)_1_Li][N(SO_2_CF_3_)_2_] SILs [24,26,33,47].

**Figure 7:**
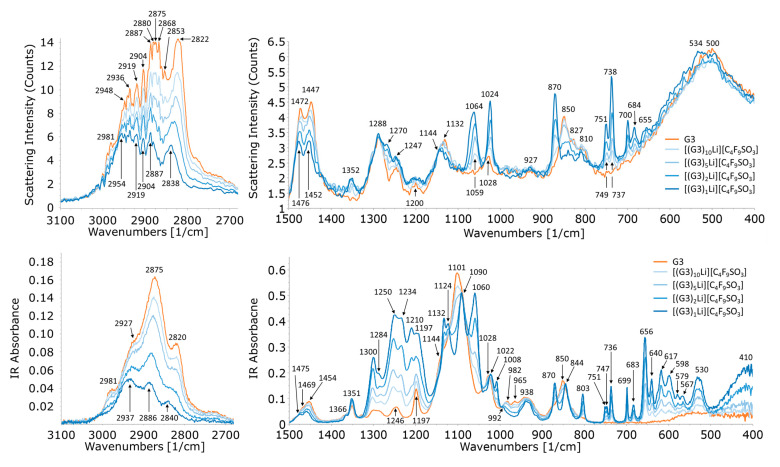
Raman (**top**) and infrared (**bottom**) spectra of [(G3)*_n_*Li][C_4_F_9_SO_3_] mixtures.

The cation–solvent interactions predicted by MD simulations may be experimentally verified with vibrational spectroscopy. Infrared and Raman spectra of [(G3)*_n_*Li][C_4_F_9_SO_3_] mixtures are given in Figure 7. A number of the G3 bands experience wavenumber shifts and loss of intensity upon mixing with LiC_4_F_9_SO_3_. For example, the 1101 cm^−1^ band, which contains large amounts of C–O–C stretching motions [40,48,49,50], red shifts to 1090 cm^−1^ in [(G3)_1_Li][C_4_F_9_SO_3_]. There are also relatively subtle changes to the CH_2_ wagging modes at 1447 and 1472 cm^−1^ as well as decreases in C–H stretching mode intensity. The latter is especially pronounced for the lower-wavenumber C–H bands. The 800 to 900 cm^−1^ region is an especially important spectral region to consider because the bands that occur here are sensitive to the conformation of the ethylene oxide backbone. G3 bands found in this region are mixtures of CH_2_ rocking, CO stretching, and CC stretching motions [50]. The characteristic way G3 molecules wrap a Li^+^ ion to accommodate tetradentate coordination induces conformational sequences in the backbone that lead to the appearance of a band at 870 cm^−1^ [31,51,52]. It is noteworthy that the 870 cm^−1^ band is present in the crystalline and solution phases of diethylene glycol dimethyl ether (diglyme, G2) and LiCF_3_SO_3_ because it establishes the G3 conformational sequence found in the (G2)_1_LiCF_3_SO_3_ crystal is also present in the solution phase [51]. Moreover, the absence of this band in the Raman spectrum of pure G3 has led several groups to use it as a spectroscopic fingerprint for the presence of tetradentate [(G3)_1_Li]^+^ complexes in liquid-phase SILs [24,31,44,46]. Observing the 870 cm^−1^ band in our vibrational spectra is compelling evidence for the presence of these species and confirms the MD simulation results.

Additional evidence for Li^+^ coordination by G3 molecules and anions is obtained from far-IR spectra of [(G3)_1_Li][C_4_F_9_SO_3_] in Figure S4. The SIL exhibits a broad IR band at 414 cm^−1^ that shifts to 438 cm^−1^ upon isotopic substitution with ^6^Li. This behavior strongly suggests that the band is best assigned as a lithium-ion “cage” mode. These vibrational modes occur when lithium ions undergo translatory motion in a cage-like environment defined by their neighboring atoms. A simplistic model for this mode views the lithium ion as a harmonic oscillator with the ligating O atoms being held stationary. This model predicts an 8% increase in the band wavenumber upon isotopic exchange, which compares quite well with the experimentally observed 6% increase.

### 2.3. Anion Dihedral Angle Distribution Analysis

Dihedral angle distribution functions for the perfluorobutyl part of the C_4_F_9_SO_3_^−^ anion are shown in Figure S5. MD simulations reveal a relatively rigid perfluorobutyl chain with SCCC torsion angles adopting a trans conformation. The CCCC torsion angle is slightly skewed from the ideal trans conformation, with a small population of gauche conformers present. The LiC_4_F_9_SO_3_ distributions are somewhat different from [(G3)*_n_*Li][C_4_F_9_SO_3_], but this is likely due to the higher temperature used in the simulation of the pure salt. Otherwise, the LiC_4_F_9_SO_3_:G3 ratio has a negligible impact on anion conformation. These MD simulation results are augmented by DFT calculations on isolated C_4_F_9_SO_3_^−^ anions. The stationary-state structures and relative energies of select perfluorobutyl conformations are summarized in Table 2. Consistent with the MD simulations, the tt conformation is the most stable among those investigated. This is followed by slightly less stable tg^±^ and g^±^t conformers (1–2 kJ mol^−1^ higher in energy).

Bands associated with the C_4_F_9_ group are challenging to assign given the potential for conformational flexibility. Therefore, a normal coordinate analysis was conducted on these five conformers to aid in the assignments of the anion’s vibrational modes. Calculated frequencies, IR intensities, and Raman activities are provided in Table S1; a subset of these results for modes particularly sensitive to the conformation of the C_4_F_9_ group are collected in Table 3. The majority of the conformationally sensitive anion modes fall between 800 and 680 cm^−1^. Excellent agreement between experimental and calculated mode frequencies is achieved with the application of a 1.03 scaling factor. The calculations reveal small frequency differences between the g^+^ and g^−^ conformers, making it impossible to distinguish these from one another in the experimental spectra. There is only one possible tt conformation, whereas the gt and tg conformations each have two uniquely different structures that produce the same vibrational spectrum. The expected intensity ratio should be tt:gt:tg = 1:2:2 if all species are present in equal amounts. However, the degeneracy of tg and gt conformers and comparable IR/Raman spectral activities will produce an intensity ratio of 1:4 (tt to the combined sum of gt and tg). The noticeably higher intensities of tt bands relative to the tg or gt bands in the experimental spectra underscore the conclusion that most C_4_F_9_SO_3_^−^ anions adopt a tt conformation.

Another perspective on anion structure comes from the disorder longitudinal acoustic mode (D-LAM), which is observed in polymeric and oligomeric C*_n_*H*_n_*_+2_, C*_n_*H*_n_*_+2_O*_n_*, and C*_n_*F*_n_*_+2_ molecules [52,53]. These modes are characterized by atomic displacements parallel to the skeletal backbone. D-LAMs gain radial atomic motions if the molecule adopts other conformations, which shifts the mode’s frequency and broadens the band. Hence, G3′s D-LAM serves as an excellent probe of molecular conformation. Calculations put the tt conformer D-LAM at 173 cm^−1^. The band wavenumber is higher when C_4_F_9_SO_3_^−^ anion adopts gauche conformations (183 cm^−1^ for tg^±^, 186 cm^−1^ for g^+^t, and 188 cm^−1^ for g^−^t conformers). Experimental data place D-LAM at 177 cm^−1^ (Figure S6). This further supports the conclusion that [(G3)*_n_*Li][C_4_F_9_SO_3_] contains a large population of tt anion conformers.

### 2.4. Ionic Association of Cations and Anions

**Figure 8:**
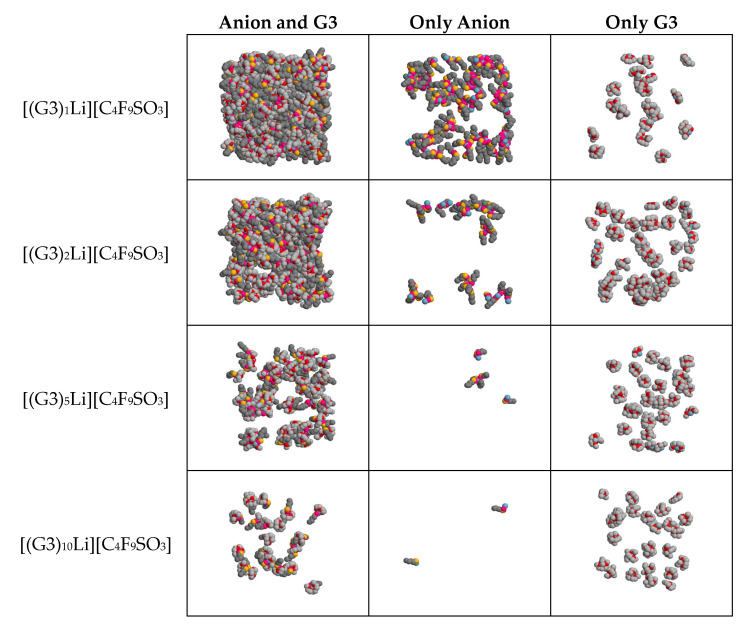

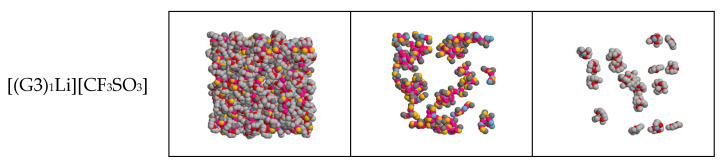
Simulation snapshots of the [(G3)*_n_*Li][C_4_F_9_SO_3_] and [(G3)_1_Li][CF_3_SO_3_] mixtures with different compositions, highlighting the Li^+^ ions coordinated by C_4_F_9_SO_3_^−^, CF_3_SO_3_^−^, and G3. The blue color represents Li^+^ ions; the colors pink, yellow, and dark grey denote the O, S, and C atoms of the anion, respectively; the colors red and light grey represent the O and C atoms of the G3 molecule, accordingly.

Competition between G3 molecules and anions for Li^+^ is apparent when the lithium complexes are discriminated by ligating molecules in the simulation boxes. The left column of Figure 8 highlights events where Li^+^ is complexed simultaneously by anions and G3 molecules. This is contrasted with events where the cations are surrounded only by anions (middle column) and G3 (right column). Even in [(G3)_10_Li][C_4_F_9_SO_3_], there is at least one anion located inside the solvation shell of about 50% of the Li^+^ ions.

The ionic association of cations and anions affects the anion’s vibrational spectrum in several ways. First, coordination can lower the symmetry of the anion, thereby splitting bands by breaking mode degeneracy. Second, coordination can directly alter the local potential energy environment in which the anion vibrates. Vibrational mode force constants are modulated by the curvature of the corresponding potential energy function; therefore, ionically associated anions may have different band frequencies relative to the uncoordinated “free” anions. Third, ionic association is often accompanied by a redistribution of electric charge throughout a molecule. This affects vibrational mode dipole moment derivatives (IR band intensities) and polarizability derivatives (Raman band intensities).

**Figure 9:**
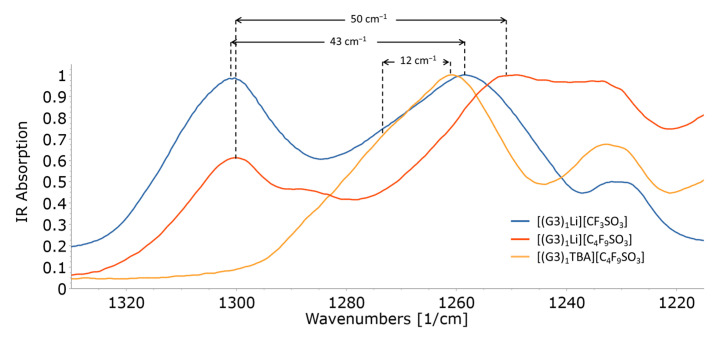
IR spectra of [(G3)_1_Li][CF_3_SO_3_], [(G3)_1_TBA][C_4_F_9_SO_3_], and [(G3)_1_Li][C_4_F_9_SO_3_].

Prior spectroscopic work on CF_3_SO_3_^−^ suggests that Li^+^ coordination occurs through the SO_3_ group [54,55]. Therefore, we begin our analysis with the antisymmetric stretching motions of the S–O bonds ν_as_(SO_3_). These bands are presented in Figure 9. [(G3)_1_Li][C_4_F_9_SO_3_] contains two ν_as_(SO_3_) bands at 1300 and 1250 cm^−1^. These bands are somewhat closer together in [(G3)_1_Li][CF_3_SO_3_] (43 cm^−1^ separation as opposed to 50 cm^−1^) and have collapsed into a single asymmetrically broadened band at 1261 cm^−1^ when Li^+^ is exchanged for the charge-protected tetrabutylammonium cation (TBA^+^). Uncoordinated CF_3_SO_3_^−^ ions have C_3v_ symmetry, and the ν_as_(SO_3_) modes belong to the doubly degenerate E irreducible representation of this point group. Monodentate or bidentate Li^+^ coordination with the O atoms of CF_3_SO_3_^−^ reduces the symmetry to C_s_, causing the ν_as_(SO_3_) mode to split into the two observed bands. The C_4_F_9_SO_3_^−^ anion’s response to coordination—from a spectroscopic point of view—is strikingly similar to CF_3_SO_3_^−^ [54]. This is because both anions experience similar Li^+^∙∙∙O–S-binding motifs. Uncoordinated C_4_F_9_SO_3_^−^ anions, however, have only approximate C_3v_ symmetry about the SO_3_ portion of the anion. ν_as_(SO_3_) degeneracy is not expected for uncoordinated anions. This clarifies why a small amount of splitting is observed in the spectra of [(G3)_1_TBA][C_4_F_9_SO_3_] even though direct cation–anion coordination is precluded by the bulky nature of the cation.

The amount of ν_as_(SO_3_) splitting might point to slight differences in the strength of the Li-anion coordinative bonds for [(G3)_1_Li][C_4_F_9_SO_3_] compared to [(G3)_1_Li][CF_3_SO_3_]. The SO_3_ symmetric stretching modes ν_s_(SO_3_) give a similar picture as the band is approximately 20 cm^−1^ higher in [(G3)_1_Li][C_4_F_9_SO_3_] compared to [(G3)_1_Li][CF_3_SO_3_]. A simple Mulliken charge analysis places slightly more negative charge on the O atoms of C_4_F_9_SO_3_^−^ (average = −0.940 for the tt conformer) compared to CF_3_SO_3_^−^ (average = −0.899). This might explain the relative cation–anion interaction strengths.

**Figure 10:**
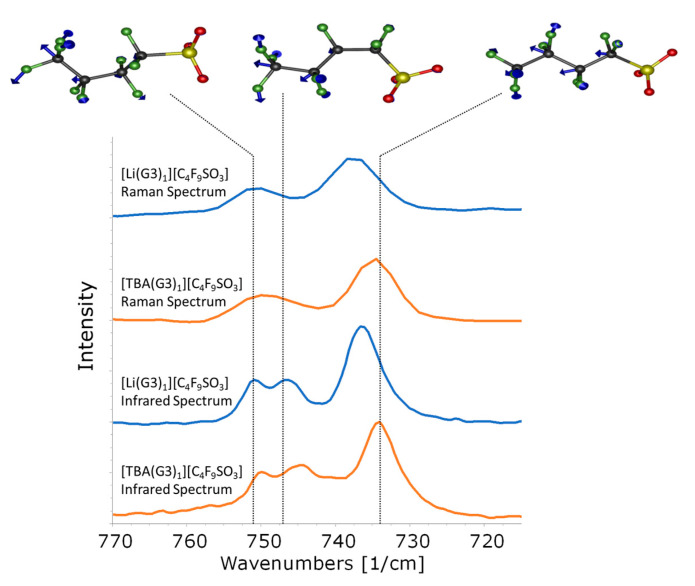
IR and Raman spectra of [(G3)_1_Li][C_4_F_9_SO_3_] (blue) and [(G3)_1_TBA][C_4_F_9_SO_3_] (orange). Spectra are displaced along the y-axis for clarity. Normal coordinate eigenvectors are displayed along the top. Vibrational mode amplitudes, which are represented by arrows on the C_4_F_9_SO_3_^−^ anion, are multiplied by 10 to aid in visualization. The eigenvectors correspond to the following conformers: tg^+^ (751 cm^−1^, **left** image), g^+^t (747 cm^−1^, **middle** image), and tt (734 cm^−1^, **right** image). Agreement between scaled DFT wavenumber calculations and experimental data is represented by the dotted lines.

We now examine anionic interactions from the perspective of the conformationally sensitive bands found in the 800–680 cm^−1^ region. Figure 10 compares the IR and Raman spectra of [(G3)_1_Li][C_4_F_9_SO_3_] and [(G3)_1_TBA][C_4_F_9_SO_3_]. Again, the TBAC_4_F_9_SO_3_ salt proves indispensable for identifying spectroscopic signatures of solvent-separated or otherwise free anions. Replacing Li^+^ with TBA^+^ causes small (1–2 cm^−1^) red shifts in the C_4_F_9_SO_3_^−^ bands while preserving the relative intensity ratios of the bands. Evidently, ionic association has a marginal effect on the conformational states of the fluorobutyl tails. Normal mode eigenvectors are included in Figure 10 to aid in the interpretation of the frequency shifts. The depicted modes are a mixture of symmetric deformations of the CF_3_ group, wagging motions of the CF_2_ moieties, and CS stretching. It may seem surprising that these modes are sensitive to cation coordination since the atomic motions involved are far from the SO_3_ group. However, this is similar to the LiCF_3_SO_3_ system where coordination triggers a redistribution of charge throughout the molecule and concomitant stiffening of the δ_s_(CF_3_) force constant [56]. It appears that a similar situation is operative in the related LiC_4_F_9_SO_3_ system.

**Figure 11:**
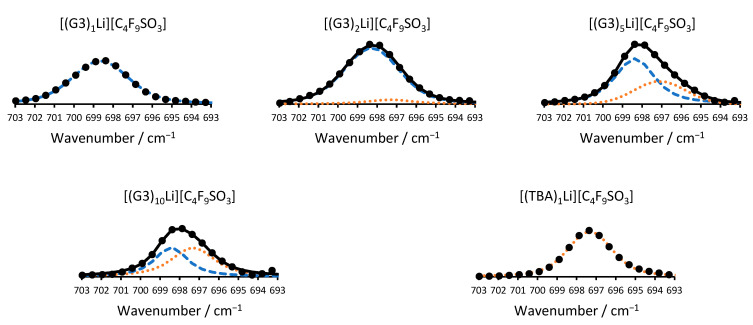
IR spectra of [(G3)*_n_*Li][C_4_F_9_SO_3_ and [(G3)_1_TBA][C_4_F_9_SO_3_]. The blue dashed and orange dotted lines represent ion-paired and free C_4_F_9_SO_3_^−^ anions, respectively. The sum of the two sub-bands for *n* = 2, 5, and 10 is shown as a black line. Experimental data are depicted as the black dots.

Ionically associated anion abundances are composition dependent. The evolution of anion speciation is analyzed through the ~700 cm^−1^ band in Figure 11. This band is selected because it may be assigned solely to tt conformers and is free from band overlap with G3. The band occurs at 698 cm^−1^ and is asymmetrically broadened toward low wavenumbers for [(G3)_10_Li][C_4_F_9_SO_3_], but the band shifts to higher wavenumbers and gains intensity when additional LiC_4_F_9_SO_3_ is added to the mixture. In contrast, the IR spectrum of [(G3)_1_TBA]C_4_F_9_SO_3_ contains a single band at 697 cm^−1^, which is assigned to spectroscopically free anions. Higher-wavenumber bands in [(G3)*_n_*Li][C_4_F_9_SO_3_] are then assigned to Li^+^···C_4_F_9_SO_3_^−^ ion pairs.

Quantitative amounts of free and ion-paired tt anions were determined by fitting the IR spectra of [(G3)_1_TBA][C_4_F_9_SO_3_] and [(G3)_1_Li][C_4_F_9_SO_3_], respectively, with single Voigt functions. Intermediate compositions of [(G3)*_n_*Li][C_4_F_9_SO_3_] were then modeled with two Voigt functions set at approximately these frequencies. Population sizes of the two species are calculated from the integrated areas of the bands. Results are summarized and compared against the MD simulations in Table 4. Discrepancies between the estimated population sizes are likely due to the effects of charge-scaling and band-fitting procedures. Reduced charges in MD simulations cause ions to be less bound to each other and have higher mobilities. Therefore, one possible explanation is that the scaled charges used in this study lead to underestimated amounts of ion pairs. An additional factor to consider is the difficulty associated with spectral deconvolution when bands are highly overlapped. The band centers differ by only 1–2 cm^−1^, which could lead to higher amounts of experimental uncertainty in the resulting ion pair population sizes. In spite of these issues, the spectroscopic and MD simulations reveal the same basic trends: C_4_F_9_SO_3_^−^ anions become highly associated when the salt content is high.

Ionic association of the CF_3_SO_3_^−^ anion is investigated through the symmetric deformation of the CF_3_ part of the anion, δ_s_(CF_3_). This particular vibrational mode is quite sensitive to anionic speciation, which has led to its frequent use in assessing cation–anion assemblages [56,57]. Table 5 contains the curve-fitting results of δ_s_(CF_3_) for [(G3)_1_Li][CF_3_SO_3_]; vibrational spectra are provided in Figures S7 and S8. The vast majority of the anions participate in either LiCF_3_SO_3_ ion pairs (70%) or [Li_2_CF_3_SO_3_]^+^ aggregates (28%). Notably, the amount of free CF_3_SO_3_^−^ is low in [(G3)_1_Li][CF_3_SO_3_].

## 3. Conclusions

The liquid structure of G3–LiC_4_F_9_SO_3_ and G3–LiCF_3_SO_3_ mixtures are evaluated with MD simulations, DFT calculations, and vibrational spectroscopy. The C_4_F_9_SO_3_^−^ anion effectively competes with G3 molecules to coordinate lithium ions. This causes a proliferation of ionically associated species and a disruption of tetradentate cation coordination by a single G3 molecule. Similar observations were noted in [(G3)_1_Li][CF_3_SO_3_], leading Mandai and coworkers [24] to classify the mixture as a “poor” SIL. We also classify [(G3)_1_Li][C_4_F_9_SO_3_] as a poor SIL given the broad similarities in structural profiles between it and [(G3)_1_Li][CF_3_SO_3_], amounts of Li^+^···C_4_F_9_SO_3_^−^ anionic association, and overall cation coordination environments. In spite of these similarities, the hydrophobic fluorobutyl side chains on the C_4_F_9_SO_3_^−^ anions exert a strong influence over G3–LiC_4_F_9_SO_3_‘s liquid structure. Anionic tails readily self-assemble into hydrophobic domains, even in the relatively dilute [(G3)_10_Li][C_4_F_9_SO_3_] mixture. This is in marked contrast to the [(G3)_1_Li][CF_3_SO_3_] system where the short CF_3_ groups are unable to drive hydrophobic structural organization. This observation provides compelling evidence that SIL liquid structure can be manipulated through structural modifications to the anion.

## 4. Materials and Methods

### 4.1. Molecular Dynamics Simulations

The molecular dynamics (MD) simulations were carried out with GROMACS 2020 [58,59,60,61,62]. Triglyme (G3) was modeled using the OPLS-AA force field [63], while LiC_4_F_9_SO_3_ and LiCF_3_SO_3_ were defined with the CL&P force field [64,65,66], which follows the same framework of OPLS. The partial charges of anion species and Li^+^ were scaled by 0.8 based on previous studies, which indicated that this procedure was necessary to obtain results close to the experimental data [67,68]. The procedure for the MD simulations involved the following steps. The initial low-density configurations built with the fftool and Packmol programs [69,70] were subjected to an MD run with 10^5^ steps of 2 fs duration in the *NpT* ensemble, using the v-rescale thermostat and Berendsen barostat (*τ*_T_ and *τ*_p_ couplings of 0.5 ps and 4.0 ps, respectively). This initial short stage brought the simulation boxes to a density resembling that of liquids. Then, annealing stages were performed at 600 K (also with a v-rescale thermostat and a Berendsen barostat). Afterwards, the systems were equilibrated at the aimed temperatures using the Nosé–Hoover thermostat and the Parrinello–Rahman barostat at *p* = 1 atm (*τ*_T_ and *τ*_p_ couplings of 0.5 ps and 4.0 ps, respectively). The production stages in the *NpT* ensemble used the velocity Verlet integration algorithm with a 1 fs timestep, a cutoff radius of 16.0 Å, smooth-particle mesh Ewald method with cardinal B-spline interpolation of order five, fast Fourier transforms with a grid spacing of 0.10 (equilibration and production), and accuracy of Ewald sum kept at 5 × 10^−6^ at the cutoff (equilibration and production). The covalent bonds with hydrogens were constrained with the LINCS algorithm (fourth-order expansion). The production stages in the *NpT* ensemble were 15 ns long, with trajectories recorded every 2 fs in the last 10 ns. Specific details of the MD simulation boxes are listed in Table 6.

### 4.2. Density Functional Theory Calculations

Structural analyses and vibrational mode calculations of the C_4_F_9_SO_3_^−^ anion were performed with the GAMESS computational package [71]. Only singlet spin states were considered. Calculations were carried out with the B3LYP hybrid functional, which uses Becke’s three-parameter hybrid exchange functional [72] and the Lee–Yang–Parr correlation functional [73,74]. Dunning’s augmented correlation-consistent basis set (aug-cc-pVDZ) was used [75]. Default Lebedev grid settings were employed in the calculation (96 radial points in the Euler–MacLaurin quadrature and 302 angular points). Pulay’s DIIS converger was used to establish a self-consistent field [76,77]. The energy error threshold for initiating DIIS was 0.5 Hartree, and the maximum size of the DIIS linear equations was ten. The Hessian matrix was computed by numerical differentiation of analytically computed first derivatives. No symmetry constraints were applied in the course of the calculations. The lack of imaginary frequencies in the normal coordinate analysis confirmed that the calculated geometries do not occupy saddle points on the potential energy landscape. Vibrational mode eigenvectors were visualized with WebMO [78].

### 4.3. Sample Synthesis

Lithium trifluoromethanesulfonate (Sigma Aldrich, St. Louis, MO, USA, CAS #33454-82-9, 99.995%), lithium nonafluoro-1-butanesulfonate (TCI Chemicals, Portland, OR, USA, CAS #131654-65-5, >95.0%), tetrabutylammonium nonafluoro-1-butanesulfonate (Sigma Aldrich, CAS #108427-52-7, ≥98.0%), and 1,2-bis(2-methoxyethoxy)ethane (“triglyme” or “G3”, Sigma Aldrich, CAS #112-49-2, 99%) were used as received. Sample manipulation was performed in an argon-filled glove box with <1 ppm H_2_O. Stoichiometric ratios of the salts and solvents were obtained from mass measurements and mixed together in glass scintillation vials by magnetic stirring. Gentle heating on a hot plate was occasionally needed to aid the dissolution process.

### 4.4. Vibrational Spectroscopy

Attenuated total reflectance Fourier-transform infrared (ATR FT-IR) spectroscopy was used to generate mid-IR (4000–400 cm^−1^) and far-IR (700–100 cm^−1^) spectra of the compounds. Mid-IR spectra were collected with a Bruker Alpha FT-IR spectrometer, which was equipped with a single-bounce diamond ATR crystal, a deuterated triglycine sulfate (DTGS) detector, and a SiC globar source. Far-IR spectra were measured with a Nicolet 6700 FT-IR spectrometer. This instrument is equipped with a deuterated lanthanum α alanine-doped triglycine sulfate (DLaTGS) detector covered with a polyethylene window and an EverGlo IR source. Samples for far-IR analysis were deposited onto a Harrick DiaMAX diamond ATR accessory. Mid- and far-IR spectra were collected at 1 cm^−1^ resolution. FT–Raman spectroscopy was performed with a Thermo NXR 9610 Raman spectrometer. Each sample was transferred to a quartz NMR tube and sealed inside an argon-filled glove box. The samples were then measured in a 180° backscattering geometry with a 976 nm excitation laser set to 1.0 W. The spectral resolution was 4 cm^−1^. All of the vibrational spectra were measured at room temperature and ambient pressure. Spectral deconvolution was performed with Fityk (version 1.3.1) [79].

## Figures and Tables

**Table 1:** Average number of Li–O connections per lithium ion in [(G3)*_n_*Li][C_4_F_9_SO_3_]. Li–O_anion_ and Li–O_G3_ denote the total number of cation–oxygen contacts between Li^+^ and the anions or G3, respectively. Li-anion and Li–G3 stand for single Li-anion and Li–G3 contacts. Li–O_total_ denotes the total number of cation–oxygen connections per Li^+^ ion. Additionally, the percentages of free C_4_F_9_SO_3_^−^, CF_3_SO_3_^−^, and G3 molecules are given.

	[(G3)*_n_*Li][C_4_F_9_SO_3_]					[(G3)*_n_*Li][CF_3_SO_3_]			
*n*	0	1	2	5	10	0	0	0	1
T/K	370	298.15	298.15	298.15	298.15	298.15	370	600	298.15
Li-O_anion_	3.75	1.93	1.35	0.98	0.60	3.73	3.68	3.21	2.02
Li-anion	3.65	1.89	1.31	0.93	0.56	3.66	3.59	3.12	2.00
Li-O_G3_		2.98	3.79	4.21	4.66				2.98
Li-G3		0.92	1.17	1.31	1.54				0.90
Li-O_total_	3.75	4.92	5.14	5.19	5.26	3.66	3.68	3.21	5.00
Free anion		4%	16%	27%	50%				3%
Free G3		15%	42%	74%	85%				14%

**Table 2:** Relative energies and torsion angles of five C_4_F_9_SO_3_^−^ conformers.

Conformation	tt	tg^−^	tg^+^	g^+^t	g^−^t
Structure	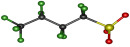	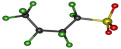	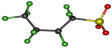	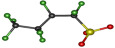	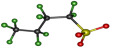
Relative Energy (kJ/mol)	0	1.79	1.82	1.52	1.54
∠SCCC	164.7°	−166.7°	166.7°	56.1°	−56.0°
∠CCCC	162.3°	−54.8°	54.8°	162.4°	−162.3°

**Table 3:** Assignments for the select vibrational mode of the C_4_F_9_SO_3_^−^ anion. Scaled frequencies (shown in parentheses) are obtained by multiplying the calculated frequencies by 1.03.

[(G3)_1_Li][C_4_F_9_SO_3_] Frequency/cm^−1^		[(G3)_1_TBA][C_4_F_9_SO_3_] Frequency/cm^−1^		Calculated [(G3)_1_Li][C_4_F_9_SO_3_] Frequencies/cm^−1^				
IR	Raman	IR	Raman	tt	tg^−^	tg^+^	g^+^t	g^−^t
683	684	681	682		662 (682)	662 (682)	666 (686)	666 (686)
699	700	697	698	681 (702)				
736	738	734	735	712 (734)				
747		745					725 (747)	726 (748)
751	751	750	750		729 (751)	729 (751)		
803	804	800	803	772 (796)				

**Table 4:** Relative population sizes of free and ion-paired C_4_F_9_SO_3_^−^ anions. MD simulation results are given in parentheses next to the spectroscopically determined values.

Composition	Ion Pairs	Free Anions
[(G3)_1_Li]C_4_F_9_SO_3_	100% (96%)	0% (4%)
[(G3)_2_Li]C_4_F_9_SO_3_	92% (86%)	8% (14%)
[(G3)_5_Li]C_4_F_9_SO_3_	67% (76%)	33% (24%)
[(G3)_10_Li]C_4_F_9_SO_3_	56% (50%)	44% (50%)
[(G3)_1_TBA]C_4_F_9_SO_3_	0% (N/A) ^a^	100% (N/A) ^a^

^a^ Not applicable.

**Table 5:** Relative population sizes of [Li*_x_*(CF_3_SO_3_)*_y_*]*^x^*^−*y*^ anions in [(G3)_1_Li]CF_3_SO_3_.

Assignment	IR Fit Wavenumber/cm^−1^	IR Band Percentage	Raman Fit Wavenumber/cm^−1^	Raman Band Percentage (Raman)
Free CF_3_SO_3_^−^	753.4	4.3%	753.4	4.6%
LiCF_3_SO_3_ Ion Pair	757.6	49.0%	757.6	44.5%
[Li_2_CF_3_SO_3_]^+^	762.1	41.1%	762.2	50.2%
[Li_3_CF_3_SO_3_]^2+^	765.9	3.0%	765.9	0.0%

**Table 6:** Details of the MD simulation boxes of the [(G3)*_n_*Li][C_4_F_9_SO_3_] and [(G3)*_n_*Li][CF_3_SO_3_] mixtures, where *N* is the number of species in the MD box for a given composition, *l_box_* is the final box length, *T_MD_* is the temperature, and *ρ_calc_* is the calculated density.

System	*N*_salt_	*N*_G3_	*l_box_*/nm	*T_MD_*/K	*ρ_calc_*/g·cm^−3^
LiC_4_F_9_SO_3_	600	0	5.368	370.00	1.768
[(G3)_1_Li][C_4_F_9_SO_3_]	300	300	5.462	298.15	1.481
[(G3)_2_Li][C_4_F_9_SO_3_]	200	400	5.513	298.15	1.313
[(G3)_5_Li][C_4_F_9_SO_3_]	100	500	5.572	298.15	1.149
[(G3)_10_Li][C_4_F_9_SO_3_]	50	500	5.440	298.15	1.077
G3	0	600	5.640	298.15	0.990
LiCF_3_SO_3_	600	0	4.191	298.15	2.112
LiCF_3_SO_3_	600	0	4.224	370.00	2.063
LiCF_3_SO_3_	1300	0	5.684	600.00	1.834
[(G3)_1_Li][CF_3_SO_3_]	300	300	5.002	298.15	1.331

## Data Availability

The data presented in this study are available on request from the corresponding author due to privacy.

## Supplementary Materials

The following supporting information can be downloaded at https://www.mdpi.com/article/10.3390/molecules29092071/s1. Figure S1: Simulation snapshots of pure LiC_4_F_9_SO_3_; Figure S2: Dihedral angle distribution functions of the G3 molecule in [(G3)*_n_*Li][C_4_F_9_SO_3_] mixtures; Figure S3: Dihedral angle distribution functions of the G3 molecule in [(G3)_1_Li][CF_3_SO_3_]; Figure S4: Far-IR spectra of [(G3)_1_Li][C_4_F_9_SO_3_] and lithium-6-enriched [(G3)_1_Li][C_4_F_9_SO_3_]; Figure S5: Dihedral angle distribution functions of the C_4_F_9_SO_3_^−^ ion in [(G3)*_n_*Li][C_4_F_9_SO_3_] mixtures; Figure S6: Raman spectra of [(G3)_1_Li][C_4_F_9_SO_3_] and G3; Figure S7: Transmission IR spectrum of [(G3)_1_Li][CF_3_SO_3_]; Figure S8: Raman spectrum of [(G3)_1_Li][CF_3_SO_3_]; and Table S1: Calculated vibrational mode frequencies, IR intensities, and Raman activities.
