# Supplementary material for: A Computational and Spectroscopic Analysis of Solvate Ionic Liquids Containing Anions with Long and Short Perfluorinated Alkyl Chains
Source: Molecules. 2024 Apr 30;29(9):2071. doi: 10.3390/molecules29092071 (PMC11085471; doi:10.3390/molecules29092071)
Supplement: Supplementary file 1 [file molecules-29-02071-s001.zip › molecules-2973012-supplementary.pdf]

A Computational and Spectroscopic Analysis of Solvate Ionic Liquids Containing Anions with Long and Short Perfluorinated Alkyl Chains

Karina Shimizu [1], Adilson Alves de Freitas [1,*], Jacob T. Allred [2] and Christopher M. Burba [2,*]

[1] Centro de Química Estrutural, Institute of Molecular Sciences, Instituto Superior Técnico, Universidade de Lisboa, Av. Rovisco Pais, 1049-001 Lisboa, Portugal; karina.shimizu@tecnico.ulisboa.pt

[2] Department of Natural Sciences, Northeastern State University, 611 N Grand Ave., Tahlequah, OK 74464, USA; allredj@nsuok.edu

* Correspondence: adilsondefreitas@tecnico.ulisboa.pt (A.A.d.F.); burba@nsuok.edu (C.M.B.)

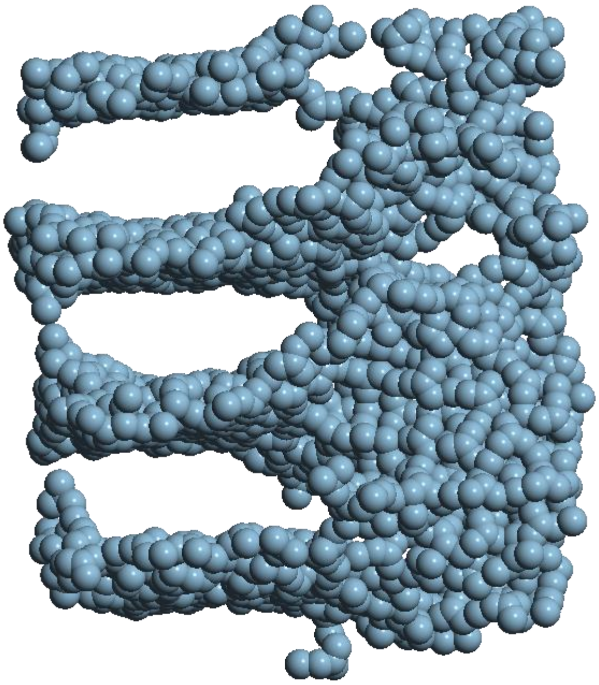

Figure S1. Simulation snapshots of pure $LiC_4F_9SO_3$ at 370 K highlighting the lamellar organization of the polar domains.

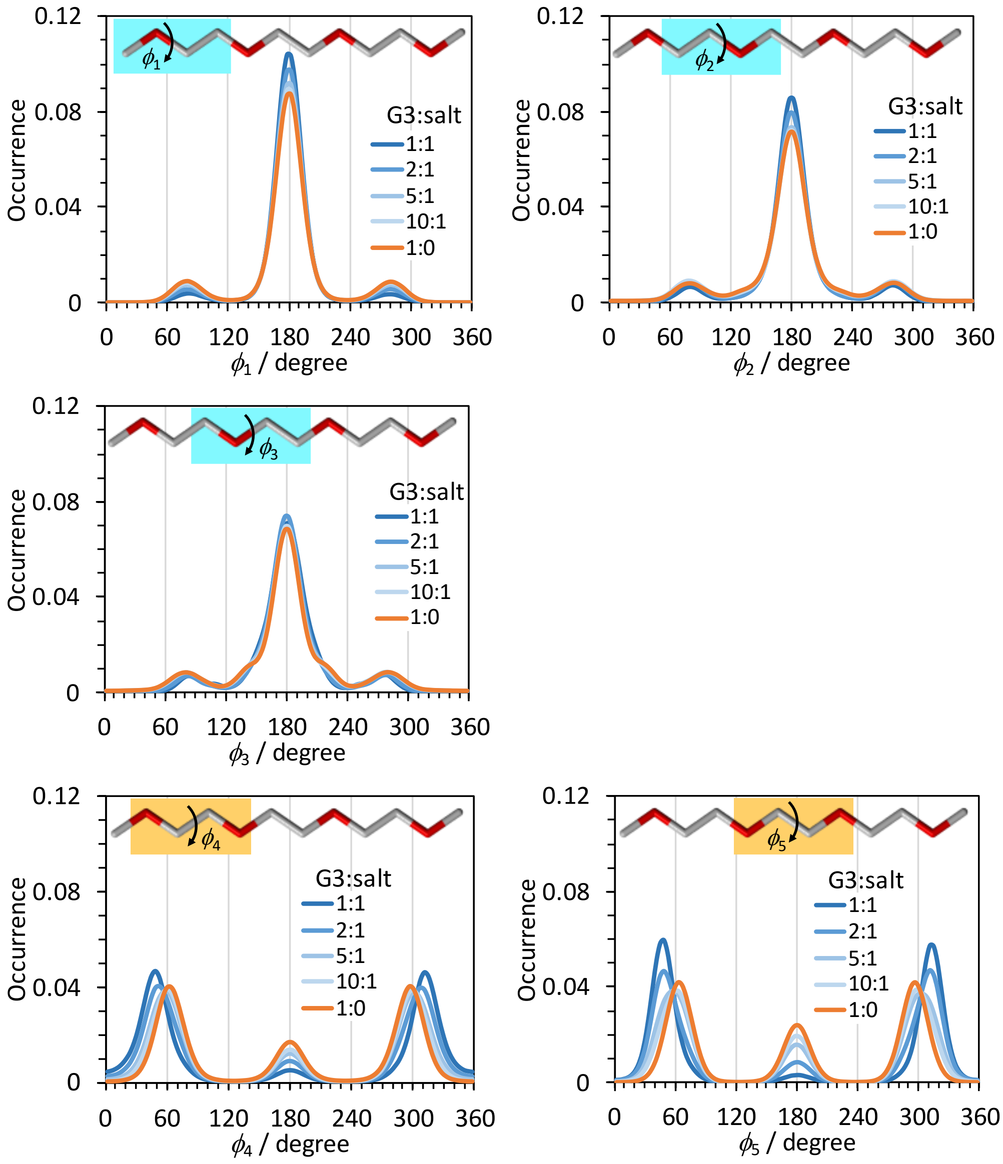

Figure S2. Dihedral angle distribution functions of the G3 molecule in $[(G3)_nLi][C_4F_9SO_3]$ mixtures. Ratios are given as G3:$LiC_4F_9SO_3$.

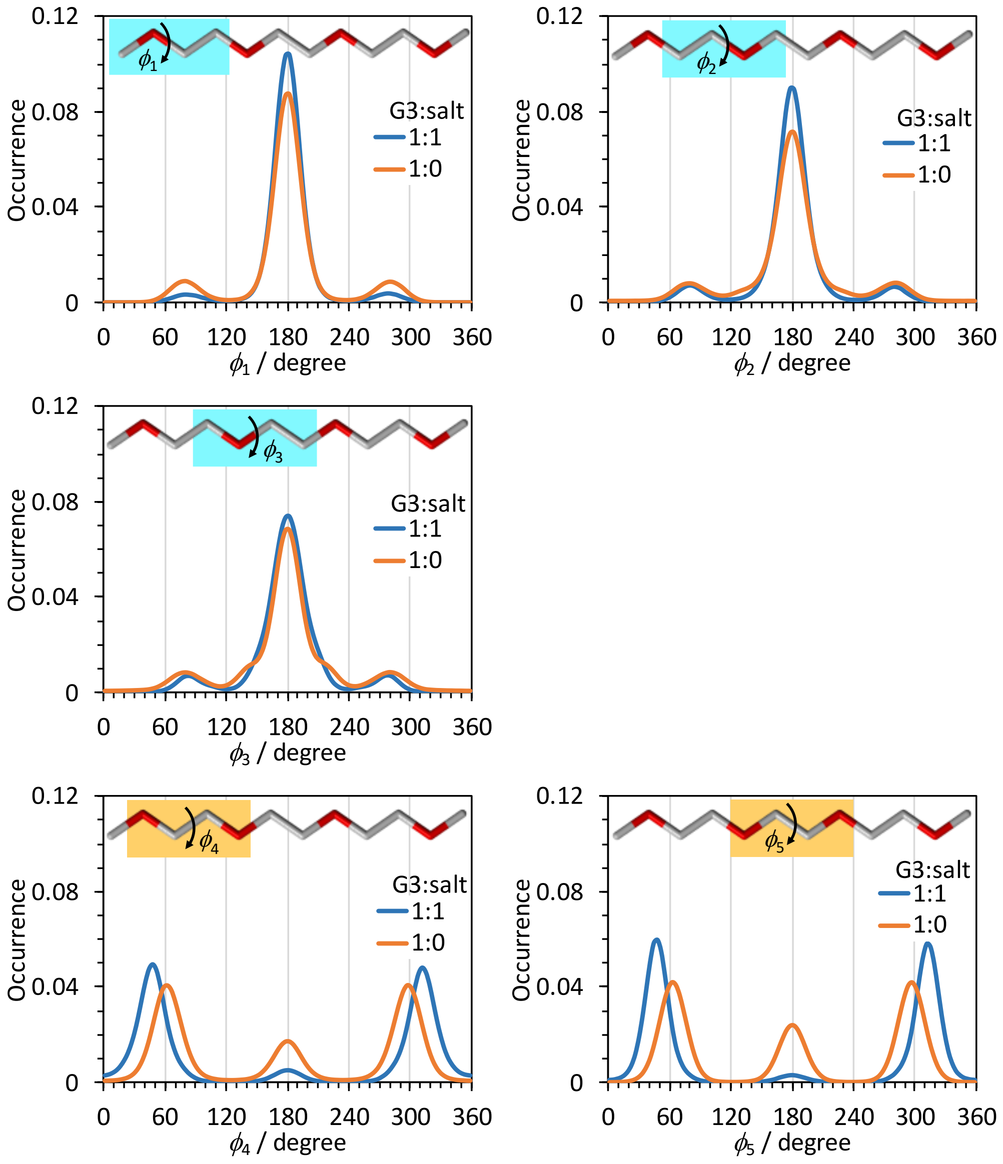

Figure S3. Dihedral angle distribution functions of the G3 molecule in $[(G3)_1Li][CF_3SO_3]$. Ratios are given as G3:$LiCF_3SO_3$.

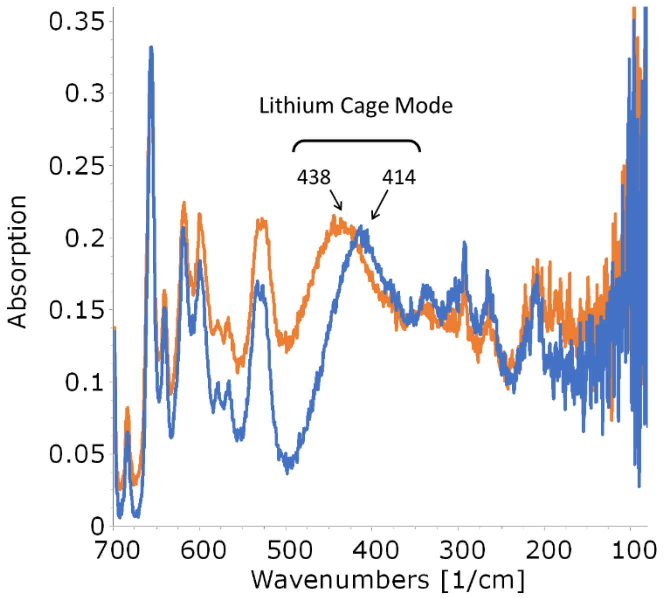

Figure S4. Far-IR spectra of $[(G3)_1Li][C_4F_9SO_3]$ (natural Li isotopic abundance, blue) and lithium-6 enriched $[(G3)_1Li][C_4F_9SO_3]$ (orange).

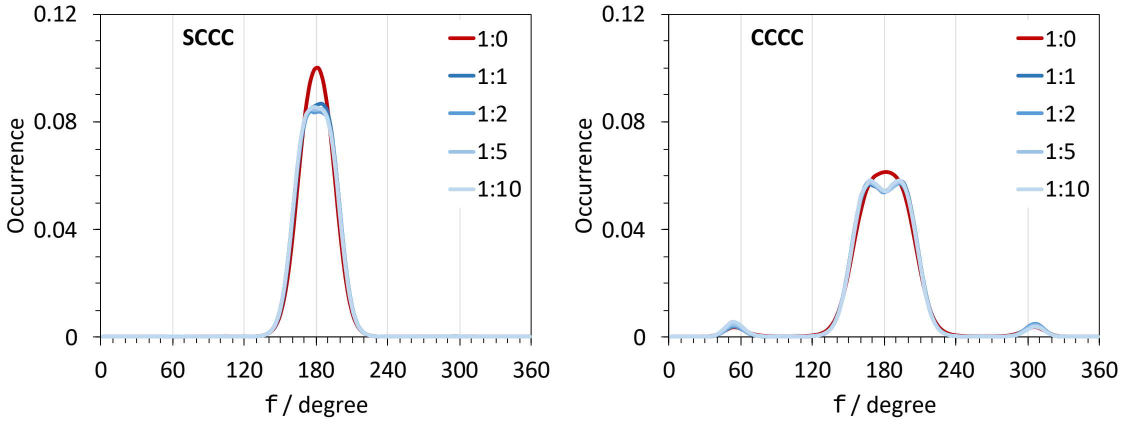

Figure S5. Dihedral angle distribution functions of the $C_4F_9SO_3^-$ ion in $[(G3)_nLi][C_4F_9SO_3]$ mixtures. Ratios are given as G3:$LiC_4F_9SO_3$.

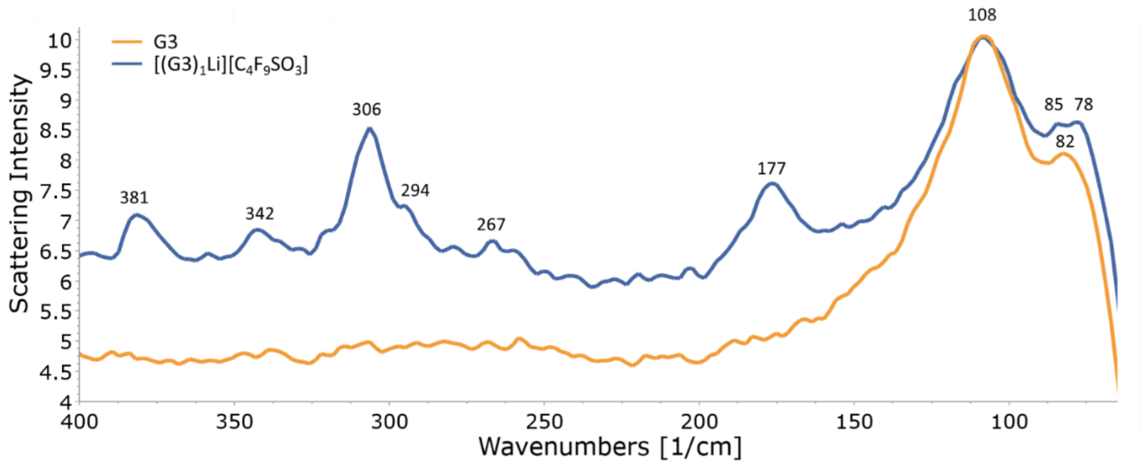

Figure S6. Raman spectra of $[(G3)_1Li][C_4F_9SO_3]$ and G3.

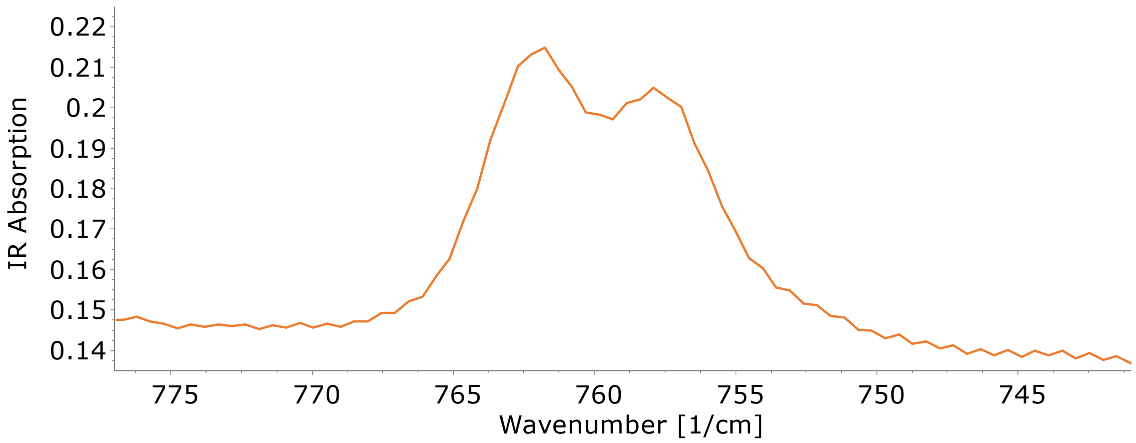

Figure S7. Transmission IR spectrum of $[(G3)_1Li][CF_3SO_3]$ measured by sandwiching the SIL between ZnSe windows. The region shown contains the $\delta_s(CF_3)$ band.

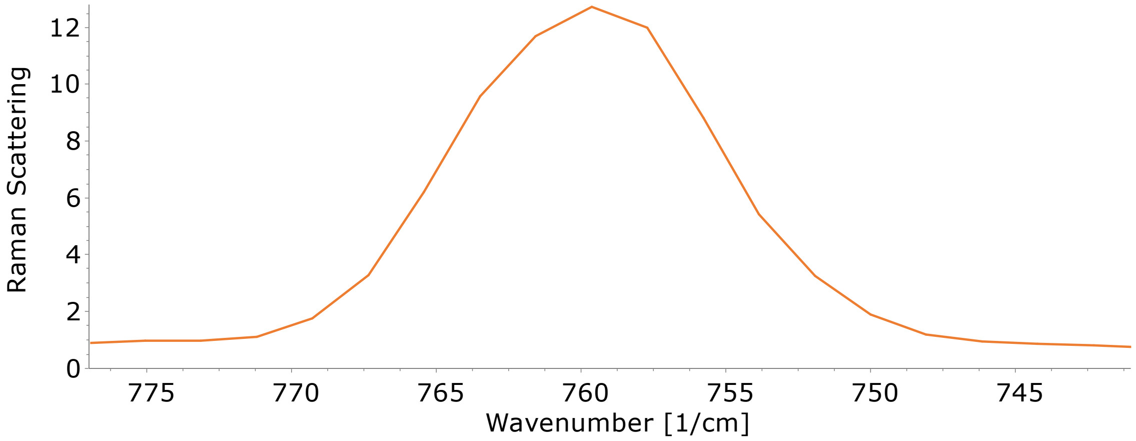

Figure S8. Raman spectrum of $[(G3)_1Li][CF_3SO_3]$. The region shown contains the $\delta_s(CF_3)$ band.

Table S1. Calculated vibrational mode frequencies (cm^{-1}), IR intensities ($km\ mol^{-1}$), and Raman activities ($Å^4\ amu^{-1}$) for the $C_4F_9SO_3^-$ anion in various conformations.

	tt Conformer			tg^- Conformer			tg^+ Conformer			g^+t Conformer			g^-t Conformer		
Mode	Frequency	IR Intensity	Raman Activity	Frequency	IR Intensity	Raman Activity	Frequency	IR Intensity	Raman Activity	Frequency	IR Intensity	Raman Activity	Frequency	IR Intensity	Raman Activity
1	28.4	0.0	0.0	33.4	0.3	0.0	32.6	0.3	0.0	32.1	0.3	0.0	40.1	0.3	0.0
2	44.1	0.6	0.8	50.1	0.4	0.0	49.3	0.4	0.0	45.6	0.5	0.1	48.4	0.5	0.0
3	56.1	0.2	0.0	63.8	0.3	0.0	63.5	0.3	0.0	66.9	0.8	0.3	69.5	0.8	0.0
4	60.0	0.4	0.4	83.2	0.3	0.0	83.4	0.3	0.0	81.2	0.1	0.1	83.3	0.1	0.0
5	97.9	1.3	0.1	106.7	0.9	0.1	106.6	0.9	0.1	106.9	1.9	0.0	109.1	1.9	0.1
6	156.1	1.0	2.4	132.9	0.9	0.7	133.0	0.9	0.7	133.3	3.0	0.1	132.9	3.0	0.4
7	167.8	0.1	0.6	177.2	1.8	1.3	177.2	1.8	1.3	180.4	0.3	1.7	182.9	0.3	0.5
8	190.8	1.0	0.1	198.6	0.3	0.4	198.6	0.3	0.3	189.2	0.7	0.4	188.5	0.7	0.2
9	213.2	1.6	1.1	221.1	1.8	0.1	221.1	1.8	0.1	217.6	1.7	3.5	217.8	1.7	0.3
10	240.0	0.3	1.3	227.3	0.5	0.8	227.3	0.5	0.8	235.1	0.3	0.2	233.8	0.3	0.3
11	241.7	0.0	0.2	242.3	0.8	1.5	242.4	0.8	1.5	243.4	0.3	0.7	245.6	0.3	0.6
12	247.9	0.3	0.5	259.5	1.8	0.9	259.3	1.8	0.9	255.2	0.0	12.0	257.8	0.0	4.1
13	284.7	0.2	4.7	285.2	0.2	4.2	285.5	0.2	4.1	283.4	0.1	4.0	283.4	0.1	2.0
14	286.8	2.2	1.5	294.5	0.3	1.9	294.5	0.3	1.9	289.0	2.6	1.1	287.4	2.6	0.7
15	300.7	0.2	3.0	309.4	0.4	0.5	309.3	0.4	0.5	311.0	0.6	3.7	311.5	0.6	1.2
16	327.7	0.2	0.8	315.9	0.6	1.1	315.9	0.6	1.1	327.9	0.3	2.6	329.1	0.3	2.6
17	353.0	0.4	0.3	345.1	0.4	0.9	345.0	0.4	0.9	336.8	0.3	0.9	336.1	0.3	0.5
18	364.0	1.3	0.3	362.9	0.1	1.7	362.9	0.1	1.7	359.9	1.3	1.2	359.9	1.3	1.4
19	372.2	0.2	1.8	379.4	1.4	0.6	379.4	1.4	0.6	381.3	1.5	0.5	380.2	1.5	0.6
20	445.1	0.5	0.2	438.0	3.4	0.5	437.9	3.4	0.5	430.4	2.2	1.0	431.5	2.2	0.3
21	494.6	29.9	1.7	497.0	12.8	0.4	496.7	12.8	0.4	493.6	22.6	1.7	494.4	22.6	0.9
22	508.4	12.5	0.5	498.9	15.8	1.4	498.7	15.8	1.4	504.6	12.3	1.9	505.0	12.3	1.0
23	516.8	9.0	1.0	516.4	10.3	1.1	516.3	10.2	1.1	515.3	2.5	0.5	517.1	2.5	0.3

	tt Conformer			tg^{-} Conformer			tg^{+} Conformer			$g^{+}t$ Conformer			$g^{-}t$ Conformer		
Mode	Frequency	IR Intensity	Raman Activity	Frequency	IR Intensity	Raman Activity	Frequency	IR Intensity	Raman Activity	Frequency	IR Intensity	Raman Activity	Frequency	IR Intensity	Raman Activity
24	542.2	9.0	1.9	537.8	13.3	2.4	537.6	13.2	2.4	525.3	12.4	3.1	527.5	12.4	2.8
25	557.7	4.3	0.5	561.5	8.1	0.7	561.5	8.1	0.7	569.3	18.5	0.7	569.9	18.5	0.7
26	581.1	67.3	1.1	575.6	24.9	1.5	575.6	25.1	1.5	584.8	6.4	1.3	584.2	6.4	1.3
27	600.8	95.8	2.0	612.2	155.3	0.9	612.0	155.2	0.9	604.2	94.1	1.0	603.7	94.1	0.8
28	627.0	56.2	2.4	633.5	12.8	2.7	633.5	12.3	2.7	622.8	61.0	2.2	623.0	61.0	2.8
29	681.2	24.4	1.2	662.4	24.5	4.5	662.4	24.5	4.5	665.8	17.5	3.5	666.1	17.5	4.2
30	712.2	34.2	7.7	729.3	20.6	9.9	729.3	20.7	9.9	724.9	37.7	11.4	726.4	37.7	9.6
31	772.4	30.6	6.5	811.6	70.2	1.5	811.7	70.5	1.5	847.4	179.2	1.1	849.1	179.2	0.1
32	979.5	25.1	24.7	977.9	12.7	23.0	977.8	12.2	23.2	947.1	56.0	16.9	946.4	56.0	9.9
33	1013.1	152.3	13.4	1000.0	269.5	15.9	999.9	269.9	15.7	999.1	57.0	19.7	998.4	57.0	24.4
34	1080.9	44.4	9.0	1076.9	46.3	1.6	1077.0	46.2	1.6	1082.4	56.3	3.3	1080.4	56.3	2.5
35	1100.5	21.0	6.6	1093.4	26.5	0.9	1093.4	26.5	0.9	1083.1	111.3	4.0	1085.4	111.3	4.2
36	1106.4	82.4	2.2	1106.7	62.3	4.9	1106.5	62.4	4.9	1113.5	21.9	4.9	1116.0	21.9	5.6
37	1141.4	65.8	0.8	1135.6	92.4	1.8	1135.5	91.3	1.8	1130.5	55.6	1.8	1131.4	55.6	0.6
38	1156.2	162.7	2.4	1145.2	105.2	2.8	1145.3	106.1	2.8	1158.6	170.1	3.9	1160.1	170.1	2.2
39	1166.0	207.9	7.2	1170.3	260.8	2.1	1170.3	261.4	2.1	1173.6	40.5	3.3	1173.7	40.5	2.4
40	1197.1	275.4	3.2	1205.8	213.2	1.0	1205.8	210.3	1.1	1189.4	371.0	1.6	1190.4	371.0	1.6
41	1228.7	288.6	11.4	1226.2	162.0	11.3	1226.0	168.2	11.3	1216.0	61.0	10.0	1219.6	61.0	2.1
42	1230.5	80.6	4.2	1236.0	400.7	7.8	1235.7	404.7	7.7	1233.3	456.5	6.0	1232.8	456.5	5.3
43	1234.3	476.7	7.6	1238.4	270.0	10.0	1238.3	263.5	10.1	1236.6	393.0	10.5	1236.4	393.0	10.8
44	1257.0	37.2	8.6	1255.9	60.6	5.7	1255.9	59.5	5.7	1270.8	17.6	5.8	1273.7	17.6	4.9
45	1326.7	77.8	8.0	1325.1	51.3	4.5	1325.2	51.2	4.5	1320.2	63.7	10.0	1324.6	63.7	5.8
